# Computer-Aided Early Melanoma Brain-Tumor Detection Using Deep-Learning Approach

**DOI:** 10.3390/biomedicines11010184

**Published:** 2023-01-11

**Authors:** Rimsha Asad, Saif ur Rehman, Azhar Imran, Jianqiang Li, Abdullah Almuhaimeed, Abdulkareem Alzahrani

**Affiliations:** 1 School of Software Engineering, Beijing University of Technology, Beijing 100124, China; 2 University Institute of Information Technology, PMAS-Arid Agriculture University, Rawalpindi 46000, Pakistan; 3 Department of Creative Technologies, Air University, Islamabad 42000, Pakistan; 4 Digital Health Institute, King Abdulaziz City for Science and Technology, Riyadh 11442, Saudi Arabia; 5 Faculty of Computer Science and Information Technology, Al Baha University, Al Baha 65779, Saudi Arabia

**Keywords:** medical imagery, brain tumor, convolutional neural network, deep learning, feature extraction

## Abstract

Brain tumors affect the normal functioning of the brain and if not treated in time these cancerous cells may affect the other tissues, blood vessels, and nerves surrounding these cells. Today, a large population worldwide is affected by the precarious disease of the brain tumor. Healthy tissues of the brain are suspected to be damaged because of tumors that become the most significant reason for a large number of deaths nowadays. Therefore, their early detection is necessary to prevent patients from unfortunate mishaps resulting in loss of lives. The manual detection of brain tumors is a challenging task due to discrepancies in appearance in terms of shape, size, nucleus, etc. As a result, an automatic system is required for the early detection of brain tumors. In this paper, the detection of tumors in brain cells is carried out using a deep convolutional neural network with stochastic gradient descent (SGD) optimization algorithm. The multi-classification of brain tumors is performed using the ResNet-50 model and evaluated on the public Kaggle brain-tumor dataset. The method achieved 99.82% and 99.5% training and testing accuracy, respectively. The experimental result indicates that the proposed model outperformed baseline methods, and provides a compelling reason to be applied to other diseases.

## 1. Introduction

**Figure 1:**
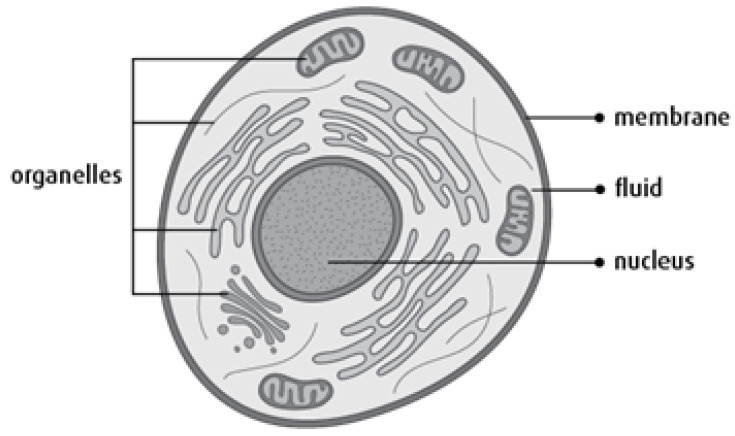
The cell.

In any living organism, cells are the main ingredient that makes up life. Cells are comprised of a cell membrane, a nucleus, and the third component, cytoplasm [1], which is the most complex organism in human beings. It is fabricated of a significant number of cells, and each of them differs in morphology. In the human body, cells continuously grow and divide in tissues. On reaching the stage of maturity, cells stop growing further and start performing their functions properly. They kill themselves when cells become damaged or their life span is completed. Due to metabolic disorders, cellular anxiety, and pathogenic incursion, cells may die during the span of development. Tissues contain natural killer cells [2], which perform such activities. These events are performed through a well-defined process known as programmed cell death (PCD) [3]. Mainly, three routes are considered under PCD for carrying out the tasks of the disposal of cells that are at risk. These paths include: pyroptosis, apoptosis, and necroptosis. The very first programmed cell death is apoptosis [4]. All of these processes are capable of sensing irritation and responding to inflammatory responses against the immune system by discharging immunostimulatory particles. Each of them is deployed with different consequences because all of them include discrete molecular processes. Those brain cells that are not proficient enough to perform phases of their life cycle in a normal fashion, form a brain tumor. Figure 1 depicts the structure of a cell. A core part of the cell is the nucleus surrounded by fluid. The exterior boundary of cells is covered by a protective sheet known as a membrane. An inside cell contains different micro-organelles including mitochondria, ribosomes, cytoplasm, DNA, etc.

**Figure 2:**
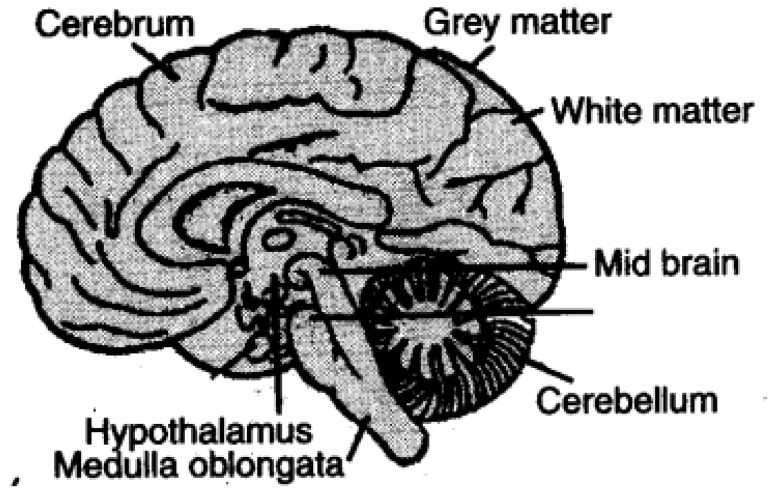
Structure of brain.

The most complex organ of the human body is the brain. It controls the whole body, including our sentiments, opinions, moods, temperature, motor action, starvation, etc. It helps all the organs of the body to coordinate [5] appropriately in order to execute their functions. At the central end of the human body, the brain is composed of a significant number of of nerve tissues. The weight of the human brain is around 3 pounds. Approximately 60% of the brain is fat and the remaining 40% is a blend of protein, salts, water, and glucose. The brain is the main part of the central nervous system. The structure of the brain includes the brainstem, cerebellum, and forehead [6]. The brain is comprised of grey and white matter, as shown in Figure 2. The cerebrum, cerebellum, and medulla oblongata are parts of the brain. The largest fragment is the cerebrum, the cerebellum is the next largest fragment, and then the medulla.

**Figure 3:**
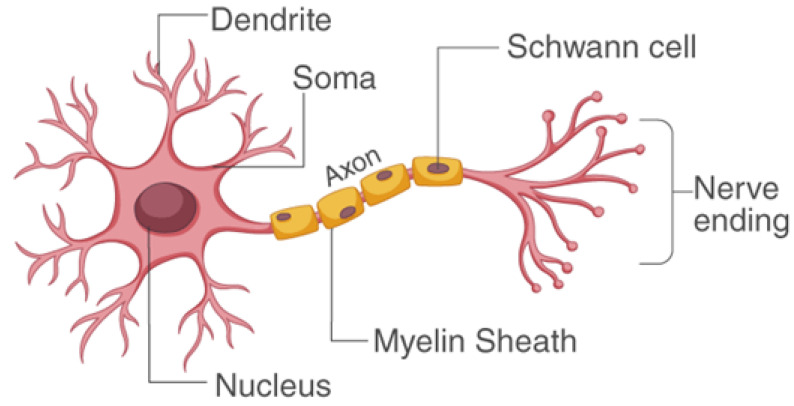
Structure of neuron.

The exterior portion, which is dark in colour and is comprised of neurons, is denoted as grey matter. White matter refers to the innermost light portion, which mainly contains axons. Sometimes, this grey matter is also denoted as the cerebral cortex. The purposeful hiearcrhy of the brain extends straight from the “uni-modal sensory cortex” into the “trans-modal association cortex” [7]. Different parts of the brain are involved in the process of memorization. Three phases are included in memorization. First is encoding, where memory is built up for any event. The second is storing a particular event in the short-term memory. Third is recalling, during which a certain event is moved from short to long-term memory. There are trillions of cells in the brain. Brain cells are known as neurons. Neurons are the chief module of the nervous system. Neurons are comprised of: sensory neurons, motor, and interneurons. They accept and transmute information from and to the brain from all parts of the body in the form of signals, as shown in Figure 3. Information from sensory receptor cells is carried from the body to the brain by sensory neurons. Then, brain information is carried to the muscles by motor neurons. Meanwhile, information between the remaining neurons of the body is transmuted through interneurons. Only one axon of a neuron, with an extensive tail-like structure, carries the nerve impulse in the form of electric signals from the body. Dendrites can be of more than one type, and accepts signals from axons.

**Figure 4:**
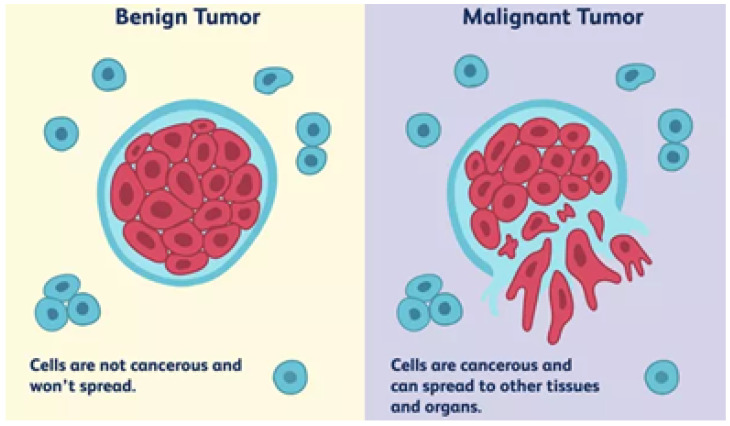
Types of tumor.

The frame of abnormal tissues, when grouped, forms into tumor. Skin, muscles, secretory organs and different body parts have the chance to be affected by a tumor. These tumors are classified into two main types, as depicted in Figure 4. One is malignant, which is cancerous. Another is benign, noncancerous [8]. Each of these tumors varies in its treatment. Therefore, before preliminary action, first of all, it is necessry to recognize the type.

Benign tumors remain at one location. They grow at these sites without changing their positions. They do not harm cells that reside near them. They are not dangerous. Once this type of tumor is disposed of, they have no chance of returning. On the other hand, malignant tumors do not reside in one location. They tend to continuously change their position along with their growth. These cells are dangerous, as they are cancerous. They harm other cells near them by invading. Therefore, they need to be treated to control them. They are treated through immunotherapy or chemotherapy [9].

**Figure 5:**
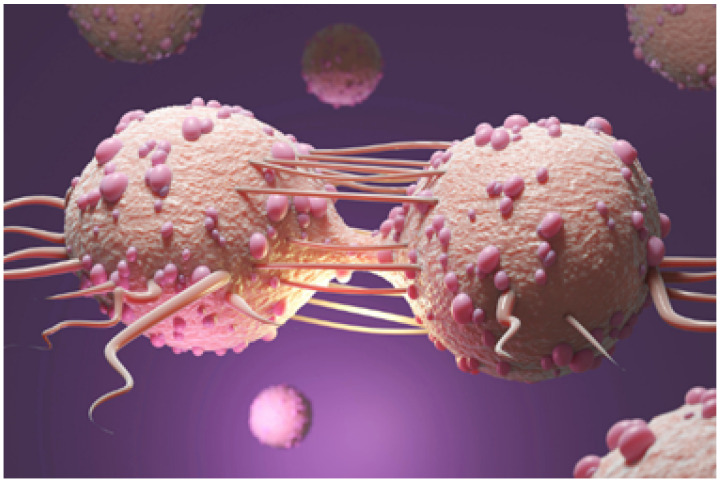
Shape of tumor.

When cells abnormally grow in the brain, they form a brain tumor. Symptoms of these tumors vary according to their size and location. Sites for the occurrence of tumors in brains include cranial nerves, meninges (membranes), pineal secretory organs, and pituitary secretors. Risk factors for brain tumors may vary. When tumors from skin cells spread to the brain, this is known as a melanoma brain tumor. Studies have revealed that some environmental reasons may pave the way for triggering tumors in the brain. Out of these, air pollution is one reason [10]. People who are exposed to radiation at their jobs have higher chances of suffering from a brain tumor. Genetic variation is another reason for its occurrence. About 5–10 percent of people with this disease are those who have a family history of brain tumors. The most important categories of tumor include: the skin, lungs, and breast, which may spread to the brain, triggering a tumor there. In 2021, around 84,000 individuals were detected to have a primary brain tumor. About 120 variants of this primary brain tumor exist. Out of them, one-third of tumors are cancerous (malignant). Tumors can be detected at any age group. In the US, more than 28,000 children are detected as having this hazardous disease. Due to primary brain tumors, approximately 18,000 persons died [11]. Figure 5 illustrate the shape and size of a tumor and how they start growing on a mass of tissues.

**Figure 6:**
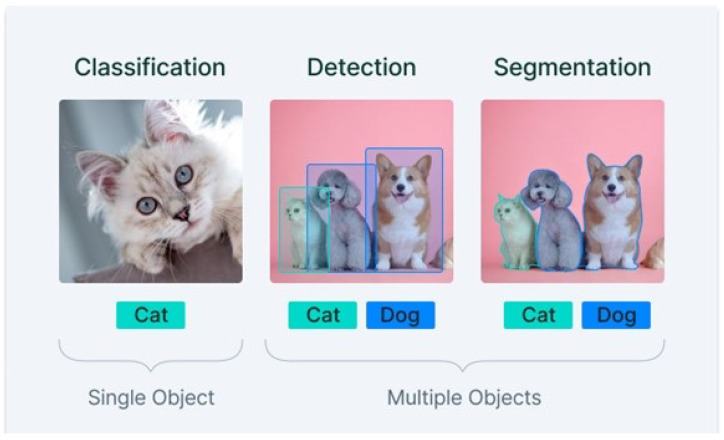
Tasks of computer vision.

The field of artificial intelligence which enables machines to see is known as computer vision (CV). CV enables machines to distinguish visual stimuli clearly [12]. These machines then process and interpret digital multimedia images and videos. After interpreting images, information is extracted to make certain decisions. Components of CV are: machines for the interpretation of scenes, a camera for capturing images, and lighting effects for illumination to better recognize objects. This CV trains machines to perform object detection through image processing. Training such a machine requires a lot of data because computer machines work consistently and make objective decisions only. CV has flourished in every field, from medical to digital marketing. The list of the various applications of CV includes road-traffic administration and self-propelled driver aid, industrial robotics and scrutiny in semiconductor unit engineering, simultaneous visualization for medical applications, and eye and head tracking for purchaser investigation [13]. Much work on CV in the field of medical science has been carried out using deep-learning techniques [11]. Some tasks that come under CV are the classification of images, detection of objects, tracking objects, and retrieving content-based images. CV uses certain algorithms to train machines on a large set of data. After training, digital images are passed on. Machines train themselves to learn by extracting information from images. Later the objects are classified on the basis of feature extraction. Figure 6 demonstrates the tasks of the classification, detection, and segmentation of images. It shows how the trained model has worked by passing images of cats and dogs to it. Digital images are processed successfully by classifying and detecting these objects by extracting their features.

**Figure 7:**
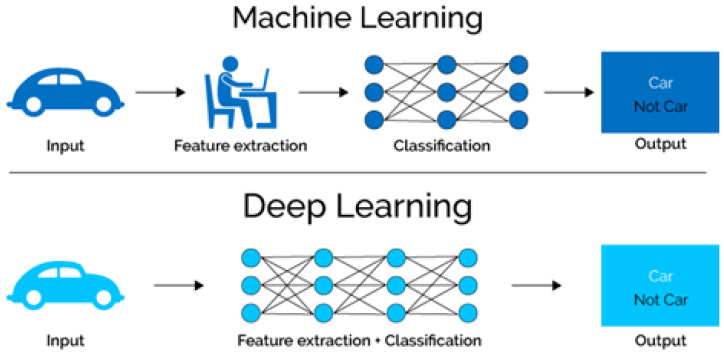
Working on deep-learning model.

Deep learning (DL) is a sub-field of artificial intelligence. It enables computational models to acquire knowledge and expand their ability to work like humans. It has multiple layers for providing abstraction and retrieving information [8]. DL is a central component of data science, including analytical modelling with facts and figures. It helps data scientists who need to carry out the tasks of collecting useful insights from huge datasets. Utilizing a huge number of datasets, deep learning achieves the best performance and accuracy [5]. It also makes the procedure of examining data effective and easier. A predominantly convolutional neural network (CNN) is implemented for the acknowledgement of patterns [14]. CNN assists in the understanding of digital images. CNN breaks down images into picture elements and allocates tabs and markers to them. Then, it classifies objects by training models based on these markers. Figure 7 shows the working of machine learning (ML) vs deep learning (DL). Utilizing ML techniques for recognizing tumors in the brain accurately is a complicated job [15,16]. For identifying features, ML techniques are deployed [17].

The physical sorting of brain-tumor MR images with similar structures or presences is a problematic and challenging task [18]. Using different imagery procedures, irregular changes in tissues of brain cells are identified at an early stage from “computed tomography (CT)” scans and “magnetic resonance imaging (MRI)” [9]. Along with the CT scan, the X-ray technique also helps in the examination [19]. MRI images are classified into grey and white matter after detection [7].

The main contribution of this study includes the detection and classification of brain tumors using a deep-learning-based convolutional neural network with an SGD optimization algorithm. Most of the previous studies have implemented CNN on a smaller dataset; while the proposed method is evaluated on a larger public dataset comprising three datasets (figshare, SARTAJ, and Br35H). The transfer-learning-based ResNet-50 is employed to extract features from MRI scan images. The detection and classification of these images are performed efficiently with better accuracy and performance using the SGD classifier with CNN. This combination of classifiers has made it possible to achieve 99.82% training accuracy and 99.5% testing accuracy using a deep convolutional neural network with stochastic gradient descent (SGD) optimization algorithm.

The rest of this paper is organized as follows. Section 2 contains the literature review on brain-tumor detection and a comprehensive analysis of other related work. Section 3 includes the proposed methodology, process for the detection and classification of tumors, as well as the proposed framework. Section 4 presents experiments and results which will contain the experimental analysis and discussion, followed by a conclusion and future work in Section 5.

## 2. Literature Review

This section highlights that detecting tumors of the brain with the help of machine-learning and deep-learning algorithms is an ongoing research area. A lot of work has already been carried out and researchers are continuously completing studies to improve this progress. The segmentation of the brain constitutes [10] a crucial part in medical training and exploration setting. A 3D deep neural algorithm based on networks was proposed for finding infected cells in the brain. Segmentation within the cells of tumors was achieved together with edema, mortification, non-magnified, and magnified tumors using MRI images. A collection of cascaded U-Nets was designed for the recognition of tumors. Along with this, Deep-CNN was made for patch-based segmentation within tumor cells. This model was used to learn the position of tumors in the brain before segmentation. The proposed model used the “BraTS-2017” challenge database as a dataset, which consisted of 285 trained subjects, 146 subjects for testing, and 46 subjects for validation. Four sequences of MRI images were made which were used by each of these subjects. These sequences include T1, T2, T1C, and FLAIR. The resolution of all the MRI data images used was set to comprehensible. Before detection, a ground-truth table was pre-processed. Using segmentation, four classes of intra-tumours were identified through the ground-truth table. depicts how the two different scales of cascaded U-Nets were used to perform the proposed work. Datasets consisted of three up-sampled and two down-sampled blocks. Using the proposed algorithm on the data set of BraTS 2017, a Dice-similarity coefficient of 0.81, 0.69 and 0.55 was achieved. The limitation of the proposed algorithm is that the machine-learning algorithm used encountered generation difficulties when they were encountered by a new dataset. Problems with deep models cause decreased performance on validation and test datasets. The class imbalance used in the dataset was another problem faced by deep models.

Sharif et al.’s recent work [20] was carried out using many procedures for classifying brain tumors using different computer-aided methods. However, the low accuracy produced by these applied methods was a significant concern. For the classification of a multi-brain tumor, a new computerized deep-learning model was proposed. That model deals with major issues encountered during classification, which include: the resemblance between different types of tumor, a highly dimensional dataset, and using a smaller number of attributes for feature extraction. The deep-learning model was applied without any pre-processing of data. The already-trained deep-learning model “Densenet201” was used and later trained for the proposed work using “deep-transfer learning”. An imbalanced dataset was used for directing training. An approach known as “Entropy–Kurtosis-based High Feature Values” was used for extracting features and the KNN was applied to hand pick the best feature out of them. Using standard deviation, the “Modified Genetic Algorithm (MGA)” was also used for picking the best features. That was carried out by finding the Euclidean distance of each attribute in the dataset. Mutation and cross-over were applied, in case the best-fit criteria was not met. Redundancy was detached from the dataset later on. After feature extraction, at the final stage, support vector machine (SVM) was utilized to research the classification of multi-class brain tumors. To reduce the computational cost of the entire work and take out information from the images in the dataset, a convolutional neural network (CNN) was utilized. From the CNN, two layers were removed and a new fully connected layer was introduced which consisted of four major kinds of brain tumor. BRATS 2018 and BRATS 2019 were two datasets used for directing this research because they are most useful for this field and are, additionally, projective. Both of them were reduced to the ratio of 50/50 for training and testing, respectively. The accuracy of BRATS 2018 was observed to be 99.7% and 98.8%, and for BRATS 2019 98.8% and 98.2% were noted. The accuracy of both of the datasets was improved as compared to their past performance. Still, some features can be considered to improve accuracy further.

Another work focused on the recognition of brain tumors in a transferable electromagnetic (EM) mental imagery system through a newly made “YOLOv3” model of the deep neural network [3]. Most often, the YOLOv3 model is utilized in recognizing objects with the best accuracy and with better quality speediness in computation. Using deep learning, the position of tumors in the head was perceived. For creating high-quality pixel imageries, a 3D portable, unidirectional, and compact imagery system with high bandwidth was cast-off. A deep neural network-based algorithm of YOLOv3 with a darknet-53 was utilized for programmed recognition of tumors in the brain with their position and bounding boxes in produced EM imageries. The Python program writing language with TensorFlow API was used for employing the algorithm of YOLOv3. An improved algorithm of the “delay-multiply-and-sum” image processing system was used to create EM images of the head. Every single image in the dataset created had a pixel power of 416 × 416. Almost 50 sampled images from diverse situations were composed. Additionally, for creating the final dataset, an image-augmentation technique was utilized. This created a set of data that was used for the purpose of testing, training, and validation later on. The total number of sample images in the dataset was 1000, out of which 800 were utilized for training, 100 for validation, and the remaining one hundred for the purpose of testing. After considering diverse datasets, the performance of tumor detection was inspected. The observed F1-Score and accuracy that were achieved by the trained dataset were 94% and 95%, respectively. For experimenting with “Non-Maximum Suppression (NMS),” a well-known technique was applied to identify the bounding boxes of target objects in an image. The YOLOv3 model was passed out with training data for about 200 epochs with a learning rate of 0.001. Improved accuracy with reduced validation loss was detected with the alteration in learning rate during the experiment. Thus, the applied model proved effective in recognizing diverse brain tumors which were dissimilar in their form and dimensions.

In another research work, the technique of deep learning was used to find a well-known kind of tumor known as a “Glioma” [21]. When comparing “low-grade glioma (LGG)” and “high-grade glioma (HGG)” the most serious and dangerous is HGG. MRIs proved beneficial for recognizing such forms of tumor in the brain region. Recent work performed was patch-based. Its limitation was that it takes more computational cost and also there is the chance of loss of information from the images. In this work, the segmentation and recognition of tumors in the head section were carried out using the feature-based extraction technique of deep learning. For enhancing the image quality taken for identifying tumor sections “Pixel Increase along with Limit (PIaL)” was used. The extraction of features from the images for segmentation was performed via a novel “Standard Balanced Digital Link (SBDL)”. After feature extraction, the best and finest attributes were found by applying the “Particle Swarm Optimization (PSO)” algorithm. After finding the optimal set of features, classification was performed. For the experiment inception, the V3 pre-trained model of deep learning was modified. Improvement in imageries in the dataset was achieved using the contrast-stretching technique. It was carried out to enhance the visibility of the target area in images, so that tumors could be recognized. The dataset used for the experiment was divided into two phases. In the first phase, segmentation was performed using the SBDL approach on the dataset of BRATS 2017 and BRATS 2018. Meanwhile, in the second phase, the classification technique was applied to BRATS 2013–2014 and BRATS 2017–2018. Both phases resulted in diverse accuracies and performances on the dataset used for validation. An experiment was conducted on the toolbox Matconvnet environment of deep learning using MATLAB 2018. The error rate for both datasets was different for selected optimal features. The accuracy achieved was more than 92% for classification. The inclusive consequences demonstrate that the presented approach is state of the art for both the arrangement and subdivision of brain tumors.

Recently, a lot of work has been carried out on the discovery of brain tumors. These works range from single-based analyses up to the techniques for image processing for finding the solution to eradicating the propagation of this disease. A vigorous technique known as image processing [22] was applied for the recognition and segmentation of tumors in the brain. The applied methodology used was to first segment past images of brain tumors and then to detect them. MRI images are used to obtain the final outcome. In phase one, these MRI imageries are first pre-processed. Then, those images are passed through a further algorithm to complete the task. The technique applied for classifying the picture element of an image is image segmentation. After obtaining pixel information, feature extraction was performed to obtain the optimal attributes of the images. For that purpose, “Discrete Wavelet Transform (DWT)” was applied. Later, to identify the performance of the algorithm, “Support Vector Machine (SVM)” was utilized. Pre-processing was performed so that each image in the dataset had the same dimension. During this, the processed image was first converted to grayscale. It becomes easier to fetch the characteristics from such images, as in RGB images, pixel color can act as noise. Resized grayscale images are converted into a binary representation. Later on, the technique of image segmentation was performed so that each image had its own unique attributes. An unsupervised technique known as K-means clustering was applied to segment the target objects in images into regions. Next, feature extraction was performed on the used dataset of MRI images. Seven key attributes that were fetched include energy, kurtosis, skewness, contrast, smoothness and RMS. Based on these features, SVM was applied to classify the tumors into benign and non-cancerous tumors. The study successfully showed that SVM is robust in the classification of tumors in the brain. Through it, the calculated performance comes out with an accuracy of 94.6%.

Özyurt et al. focused on finding brain tumors through super-resolution (SR) [23], which has been a challenging problem recently for too many years. Image pixel quality is enhanced through super-resolution. Using it for MRI images made it helpful for extracting useful information from images by making them more observable and vibrant. Therefore, through their borders, tumors in images will become more visible so that a tumor can be easily recognized by further passing through an algorithmic process. They proposed the use of super-resolution with the fuzzy C-means clustering technique and using machine-learning algorithms for the identification of brain tumors. The proposed work was carried out using the “DICOT format MRI images”. These images were transformed from having a lower pixel value into high-quality images through super-resolution. These images were then passed through image-processing techniques. Firstly, images were pre-processed. Later, for the extraction of features, the “SqueezeNet architecture” related to CNN was deployed. Segmented images coming out with the finest features after extraction were actually greyscaled images, which were converted from the RGB format during pre-processing. The dataset used included 50 malignant and 50 benign tumor images. These segmented images were then passed through an “extreme learning machine (ELM)” for classification. The “Cancer Genome Atlas Glioblastoma Multiform (TCGA-GBM) database” was used, which includes around 500 sampled images of diverse kinds of cancers collected worldwide. The performance and accuracy rate of the ELM model used for classification was based on the number of neurons that were present in its hidden layer. Along with the learning rate and activation function: “tribes”, “sin”, “sig”, “radbas”, “hardline”, and “lin” was also considered. The most suitable activation function out of them in brain tumor identification was “sigmoid” and the total number of neurons was around 1500. The proposed model proved helpful in the identification and removal of a segmented tumor in the brain through the FCM-SR algorithm. The study also showed that the performance of the model with FCM-SR was 10% more than the previous work involving the detection of the same tumor with (FCM) only. The performance of the model was higher, with an achieved accuracy of 98.33%.

Some other work was carried out to detect asymmetrical tumors [24] in the brain, which is a difficult task. The proposed model was based on four phases, which include: fetching features, selecting the finest attribute for classification, positioning, and subdivision. MRI images, if used without pre-processing, can be noisy in different forms such as instability in compelling pitch loops and image procurement. For reducing such noisiness from images and improving pixel quality, a “homomorphic wavelet filter” was applied. After the phase of pre-processing, suitable attributes were fetched through a “non-dominated sorted genetic algorithm (NSGA)” using a pre-trained model, “Inception V3”. These selected best attributes were, later on, passed on for classification. After that, slices of classified tumors weer passed to the YOLVO-V2 model to identify the position of the tumor area in the head section. After localization, images were passed on for segmentation of real tumor sections through the “McCulloch’s Kapur” selective information approach. The applied technique was conducted using three dataset databases. The proposed technique was evaluated on datasets from BRATS 2018 to BRATS 2020. Each dataset included a diverse number of confined images. Utilizing these images, four experiments were performed. Each came out with a different outcome. The second experiment achieved the result that the SVM classifier was the best in performance of all the used algorithms. After conducting the whole proposed research, it was found that ACC is the overall best classifier. Therefore, it was proved that, through this work, tumors were classified correctly. Prediction scores for all four phases were greater than 0.90. Tumor classification and segmentation were perfomred effectively. In the future, research conducted for the identification of tumors can be carried out through quantum-computation algorithms.

On the critical disease of brain tumors, Sharif et al. proposed a framework [25] for the identification of tumors at an early stage. According to him, if brain tumors are not treated early, the tumor may progress to a cancerous stage. Therefore, in this proposed research, at the initial phase, the “brain surface extraction (BSE)” approach was utilized to eliminate the skull. The imagery of that eliminated skull is then processed for the segmentation of tumors through the “particle swarm optimization (PSO)” technique. Pre-processed grayscale binary images are fed into the genetic algorithm (GA) for extracting optimal features for selection. Later, for classification, algorithms such as ANN, and SVM are used. Easily available databases of BRATS 2018 and RIDER were utilized for experimenting. The outcome was 99% performance with the proposed model. Another work that contributed to brain-tumor recognition used computer-vision techniques [26] along with machine-learning algorithms. The author proposed the computational framework because manually performing this task may be subject to human error during identification. The convolutional neural network deep-learning technique was used for this case, to obtain the best results. Two labels were used for the classification of the final result. One was “Tumor Detected” and another was “Tumor Not Detected”. The dataset of MRI images was downloaded and unzipped to pass through the CNN model with three layered architectures. Keras and the library of TensorFlow were used to train the model for up to 35 epochs. Before finishing with the dataset, computer-vision techniques such as pre-processing, and extracting features were performed. After experimenting, the model worked accurately with a performance of 96.08%. Further, while identifying tumors in the brain, along with CNN, a neutrosophic set of rules can be used.

Visual interface systems [27] can easily classify images into the category of tumor or not tumor by visualizing brain waves efficiently. However, practically, it is a difficult task to deploy. Therefoer, the author proposed a novel framework to perform this task more precisely with higher performance. Motor images are used for this study utilizing “electroencephalography (EEG)” motion to make it work more practically and more accurately. A well-known predictor, “OPTICAL”, was used with the long short-term memory (LSTM) algorithm of machine learning to obtain improved and enhanced images. To achieve a better performance, a regression-based approach is used along with an SVM classifier. The dataset used for performing the research was taken from two well-known databases. “BCI Competition IV” and “GigaDB” were the datasets. Through these, the OPTICAL predictor classified the motor images efficiently with better accuracy. The error rate observed during the experiment for both datasets was: 2.07 percent for the GigaDB database and 3.09% for the BCI Competition IV database.

In another work, it was mentioned that recent work focused on using surgical techniques for the treatment of tumors found in the brain. Therefore, they proposed a computational framework model of deep learning [15]. A newly proposed model of CNN known as “BrainMRNet” was utilized. The framework was built based on attention segments and a hypercolumn approach. BrainMRNet was utilized for pre-processing at an initial stage. Later on, the method of image augmentation was performed by an attention segment for each pre-processed image. Through this, important features from the image were fetched and then pass on to layers of the convolutional network. A hypercolumn approach of the BrainMRNet model fetched the important attributes from each layer of the network and maintained an array to store information. From the array, the finest and most optimal features were later selected. The dataset used for carrying out research was made up of easily available MRI imageries. These images were regarded as two labels. One is a normal image and the other included a tumor. Images were transformed into JPEG images. The conducted research depicts the comparison between the proposed model and old CNN models, which includes: VGG-16, GoogleNet, and AlexNet.

BrainMRNet model proved more effective in identifying tumors and came out with a performed accuracy of 96.05%.

Windisch et al. [28] focused on the advancement of previous methods used for brain-tumor recognition in a well-organized manner. They proposed a new approach known as the “correlation learning mechanism (CLM)”. CLM, along with a convolution deep-learning network, trained the model for identification. CT-scanned images wee rused as the dataset. CNN was trained through a thresholding approach and an algorithm known as an artificial neural network (ANN). Around 64 squared fragments of CT brain images were used as input data. This dataset was taken from the Kaggle repository specially designed for carrying out experiments on brain-tumor identification. It includes images of CT scans for both healthy and normal patients. After the pre-processing of images, they were passed to the model. The tumor was detected successfully with the CLM model. Observed accuracy was 96 and with 95% recall and precision. For the detection of tumors in GLIOMAS, a newly created deep-learning framework is known as “DeepSeg” [29]. The BRATS 2019 dataset was used for segmentation and effectively proved the relative performance of applying a variety of deep-learning models.

A “hybrid manta ray foraging optimization” approach was proposed for selecting optimal features. CNN was deployed for the identification of tumors. The dataset consisted of (a) normal Images, (b) benign tumors, and (c) malignant tumors. A study was conducted to compare between existed models of machine learning and proved helpful in the recognition of tumors in the brain with better efficiency and accuracy. Another work used CNN, DNN, and KNN [30] for identifying tumors in the brain with high accuracy and with less computational cost. The same was carried out for predicting the accuracy of SVM in [31] and the model of a neural network in [32].

Table 1 summarizes the different research work suggested recently in the area of recognizing tumors in the brain. Each study depicts different results using different techniques, approaches, and their own created proposed model for carrying out their research. Diverse databases with MRI images as a dataset were utilized to conduct experiments. The most used database was found to be “BRATS”. In addition, most of the classifiers observed for performing research were: SVM, ANN, CNN and PSO. Each algorithm came with different performances and accuracies. The final observation is to use a CNN deep-machine-learning classifier for identifying tumors, as it performs well in most of the studies giving fruitful results.

## 3. Proposed Methodology

**Figure 8:**
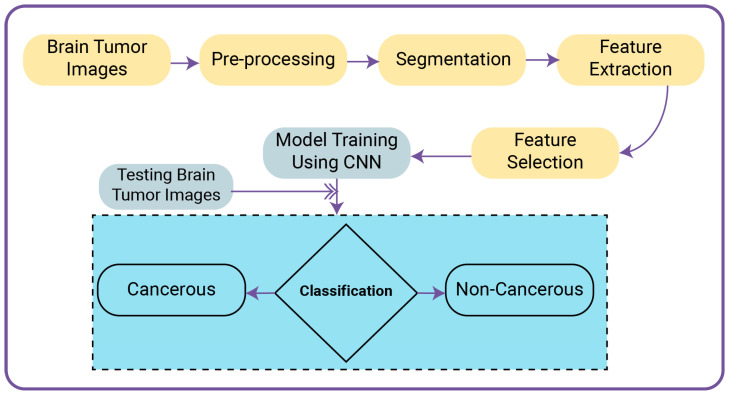
Proposed model for classification.

This section introduces the proposed framework for predicting brain tumors from the imagery data of patients with a tumor and those without. It utilizes the dataset of brain tumors taken from Kaggle. Firstly, the data is gathered. Data pre-processing is performed next, to clean the data through noise reduction, and the image pixels are converted to floating point/decimal type. Later, the pixel values of images are reduced. After that, feature extraction is performed. The working of the proposed model is shown in Figure 8.

To conduct this proposed research work, a model is trained through a supervised machine-learning algorithm known as a “Convolutional Neural Network (CNN)”. The model is trained on provided training data of brain tumors. Model, after training, classifies the provided tested brain-tumor images into cancerous and non-cancerous labels. The algorithm of the proposed framework is given below:

Recently, very little work has been carried out using MRI brain-tumor dataset of Kaggle and optimization algorithms used were ADAS, PSO, ADAM, etc. Along with the Kaggle dataset, this work will use SGD optimization algorithm. Firstly, CNN will be applied, and then an optimization algorithm (see, Algorithm 1) to gain effective results.

**Algorithm 1:** Proposed methodology algorithm

**Algorithm brain-tumor detection**
**INPUT:** MRI scanned brain tumor Image**OUTPUT:** Detection (Cancerous/Non-Cancerous)
**1.**

    **Begin:**

**2.**
    Collection of brain-tumor imagery data.
**3.**
    Combine data.
**4.**
    Apply pre-processing techniques
    **a.** Noise removal
    **b.** Segmentation
**5.**
    Apply image-processing techniques
    **a.** Feature extraction
    **b.** Feature selection
**6.**
    Train the model using CNN algorithm    **a.** Apply model    **b.** Apply optimizer and loss function
**7.**
    Analyze result after classification
**8.**

    **End**


## 4. Materials and Methods

### 4.1. Supervised Learning

A subordinate category of artificial intelligence is known as supervised machine learning. Supervised learning makes use of a labelled set of data for training machines [30]. It uses the training data to train a model to achieve the expected outcome. Training data is comprised of input and accurate outputs, which the model uses to learn with the passage of time. The accuracy of the algorithm is calculated through the loss function and is continuously adjusted until the error is minimalized, as given in Equation (1) [32].

(1) Y=f(x)

An algorithm is used to map the function from input to output values, where ‘*Y*’ is the output variable, ‘*X*’ is the input variable and ‘*f*’ is the mapping function. The mapping function is adjusted in such a way that when new input values (*X*) are entered for the data, it gives out the desired predicted outcome (*Y*). Supervised learning is classified into algorithms of “classification” and “regression”. With the help of past experience, it optimizes the performance of the model.

### 4.2. CNN Architecture

**Figure 9:**
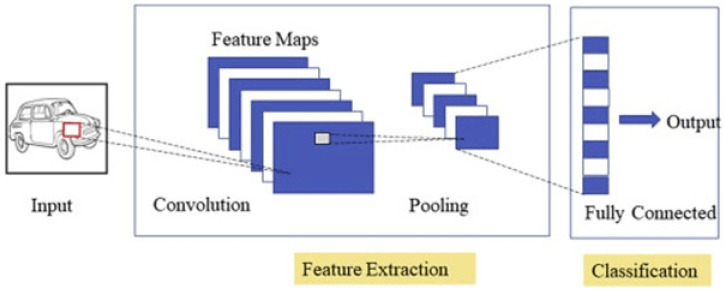
CNN architecture.

The very common deep neural network [11] and a type of feed-forward artificial neural network are CNN. It has four layers which include: “Convolution”, “ReLU Layer”, “Pooling” and “Fully Connected”, as shown in Figure 9.

When applying CNN, within an image, a filter is moved to every possible position in the convolutional layer which consists of several kernels (see, Algorithm 2). Then, “Rectified Linear Unit (ReLU)” performs its work by removing the negative values from the filtered images. Each negative value is replaced with zero. Then, pooling helps to diminish complexity by shrinking down an image. The number of filters is not affected by pooling. Actual classification takes place at the fully connected layer. The input images are passed through a trained model of CNN which are classified into some predicted outcomes. CNN shows the best accuracy in resolving classification problems with its excellent performance. It is considered a powerful tool for executing tasks related to the classification of images, image processing, and segmentation through the use of computer-vision techniques.

**Algorithm 2:** Proposed CNN architecture       **Input:** An input signal is given to CNN in form of an image or pattern.       **Output:** Classified resultant output.     **1. **
For (i=0; i<L; i++) {     **2. **
For (m=0; m<M; m++) {     **3. **
For (n=0; n<N; n++) {     **4. **
Sum =bias[i];     **5. **
For (k=0; k<K; k++) {     **6. **
For (s1=0; s1<S1; s2++) {     **7. **
Sum+-weight[k][i] [s1] [s2] *input[k][m+s1] [n+s2];     **8. **
}}}     **9. **
Output [i][m][n] =activation_func(sum);     **10.**
}}}

## 5. Experiment and Results

This section describes the process of experiments alongside the results of the proposed methodology. Once the data is cleaned, it is ready to use for classification purposes. The deep convolutional neural network is used to generate a classification model.

### 5.1. Dataset

**Figure 10:**
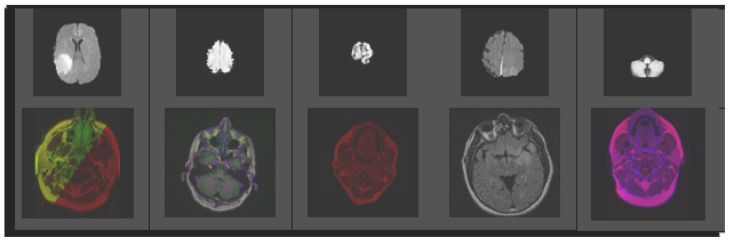
Sample images of Kaggle brain tumor dataset.

The dataset of brain tumors used for this research was taken from Kaggle. About “3762” MRI imageries were used for the classification of brain tumors. This dataset is divided into training and testing data. The dataset used for learning is termed training data and the dataset used for testing is known as testing data. Training and testing data are divided into 80 and 20 ratios, respectively. The dataset holds 2079 non-tumor images and 1683 tumor images. Here is given (Figure 10) a small glimpse of data used for experimentation purposes.

### 5.2. Prepossessing of Dataset

After the assemblage of data, it is refined through the procedure known as “preprocessing”. It is a complex and difficult task and, occasionally, this segment can take more than half of the time that is required to resolve the whole problem. It is mandatory to clean the data before employing it for training the model because most of the data gathered are not in the usable format at the time. Therefore, it needs to refine the data by preprocessing it and filtering out the data intended to be useful for classification purposes.

Data sometimes is not in a comprehensive form; to manage this issue, data is made complete through the attributes of interest that are necessary to carry out certain tasks for resolving the problem. Old traditional techniques previously used to filter data had some problems [24], including feature engineering. Before passing the dataset to any algorithm, it is cleaned by eradicating the attributes and terms that are not related to the process of classification [1]. Through this stage, data that is missing is occupied and made free of redundancy and data also comes in a suitable format.

### 5.3. Tools and Techniques

For conducting experiments, the model was created on a platform known as “Google Collaboratory”. Through it, a model was built using CNN known as “ResNet-50”. It is one of the best-known models of deep learning utilized for classifying images effectively. The residual neural network of CNN is 50 layers deep and is utilized to construct an effective model by calling the sequence model. Different libraries from Python used in model construction were TensorFlow, Keras, etc. The loss function used for the supporting model was “Binary Cross Entropy”. The optimizer algorithm used for optimizing the model’s performance was “Stochastic Gradient Descent (SGD)”.

### 5.4. Performance Matrix

Table 2, below, shows the performance metrics used to evaluate the performance of the proposed methodology. It is the confusion matrix of 2 × 2 used for plotting the diversity between the proposed values of datasets and the predicted values estimated by the models for making different assessments. The schematic of the confusion matrix is shown in Table 3.

- *Accuracy*: It is the number of correct predictions in the dataset to the total number of given inputs and is collected by Equation (2).
  (2) $$Accuracy=\frac{TP+TN}{TP+FP+TN+FN}$$
- *Precision*: It can be defined as the number of correct predictions to the total number of inputs. The *precision* can be calculated using the following Equation (3).
  (3) $$Precision=\frac{TP}{TP+FP}$$
- *Recall*: It can be defined as the correct prediction of class to the total number of inputs of that class. *Recall* can be calculated with the help of Equation (4).
  (4) $$Recall=\frac{TP}{TP+FN}$$
- *F*1-*Score* It is difficult to decide whether high *precision* or low *recall* is better when comparing different models. *F*1-*Score* combines both the *precision* and *recall* to calculate results. Equation (5). shows the *F*1-*Score*:
  (5) $$F1-Score=\frac{Precision*Recall}{Precision+Recall}$$

According to the proposed methodology mentioned in Section 5 and the experiment conducted on the data, the performance of the system is evaluated in terms of *accuracy*, *precision*, *recall*, and *F1-Score*.

### 5.5. Optimal Algorithms

**Figure 11:**
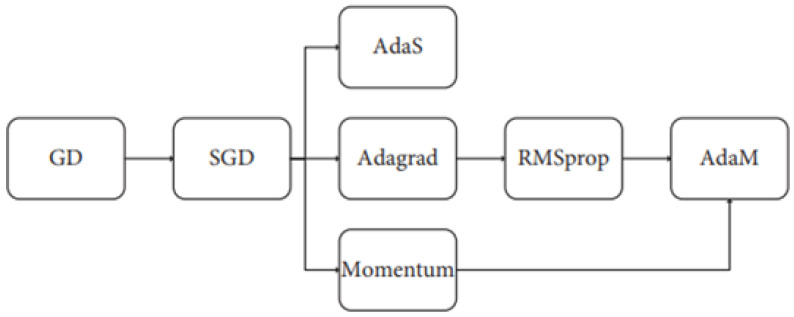
Optimization classifier.

Optimization classifiers are the main core for constructing a model through a neural network. The model learns from the input data, initializes weights, and makes an optimal prediction. The most commonly used optimization algorithms include: SGD-Stochastic Gradient Descent, GD-Gradient Descent, ADAM-Adaptive Moment Estimation, Momentum, RMSProp, AdaGrad-Adaptive Subgradient, and ADAS-Adaptive Scheduling of Stochastic Gradients. The schematic of an optimizer algorithm is shown in Figure 11.

Figure 11 shows all the optimization algorithms. SGD was picked for conducting this research because it is better than all the other classifiers in terms of having: “high generalization ability”, “quick convergence” and “high accuracy”.

### 5.6. Experimentation

**Figure 12:**
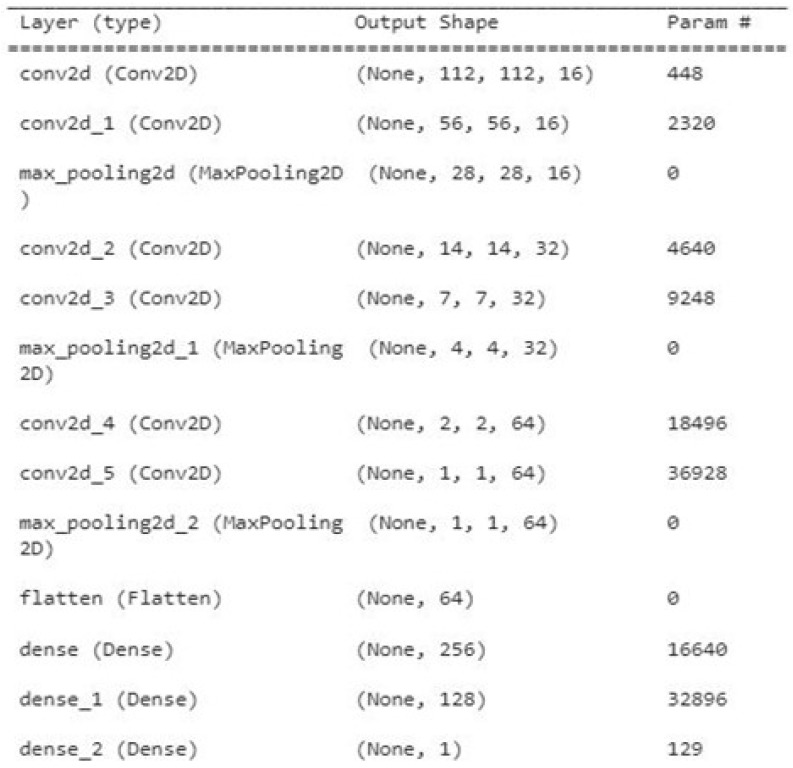
Model summary of the proposed model.

**Figure 13:**
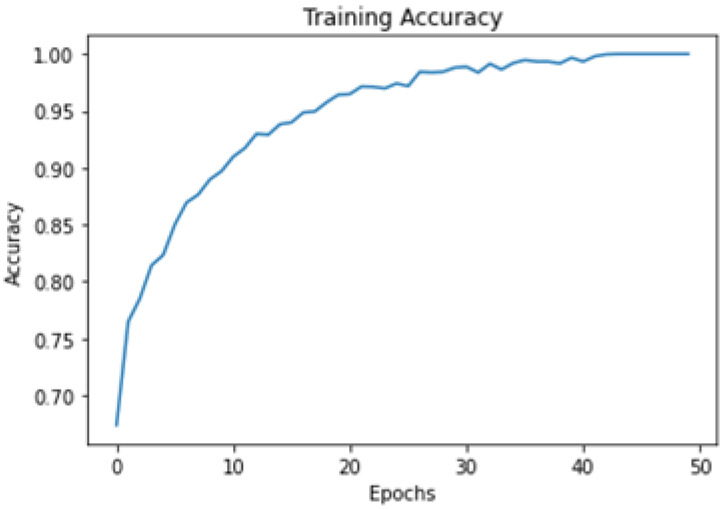
Training accuracy per epoch.

**Figure 14:**
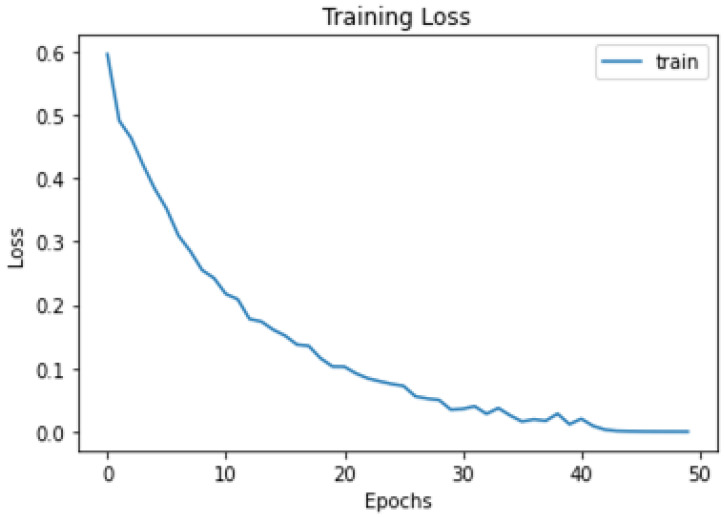
Training loss per epoch.

All the images were resized to 244 × 244 pixels from an original size of 1440 × 1440 to be used further for training and testing purposes. The model was trained with the help of an SGD optimizer along with a learning rate of 0.01 and binary cross entropy loss function. The learning rate helps sort out convergence problems. The model was trained for up to 50 epochs with a batch size of 10 and imagenet weights. The model was successfully trained on the given dataset with a training accuracy of 99.82%. A total of “753” images was used for the testing model and the accuracy achieved during the testing phase was 99.5%. The model summary of the proposed model is given in Figure 12. The graphical representation of accuracy and loss during model training is shown in Figure 13 and Figure 14, respectively.

We presented a basic CNN architecture comprised of seven layers with various hyperparameter tunings for brain-tumor detection and retained the batch size of 32 which showed optimal performance. The convolution layer has a 5 × 5 filter size followed by a pooling layer with a 2×2 filter size and stride of 2. The penultimate layer is fully connected and we applied a dropout of 0.3 to only this layer. In the final layer, we opted for a softmax output layer with ‘n’ classes. After the entire optimization process, we present the hyperparameter configurations in Table 2, which show the best possible results for brain-tumor detection.

There are various machine-learning and deep-learning models and their variations were tested in order to achieve better performance for brain-tumor detection, as depicted in Table 4. It can be evident from the results that the proposed method with the SGD optimizer outperformed the baseline method in terms of various performance metrics, i.e., F1-Score, precision, recall, and accuracy

**Figure 15:**
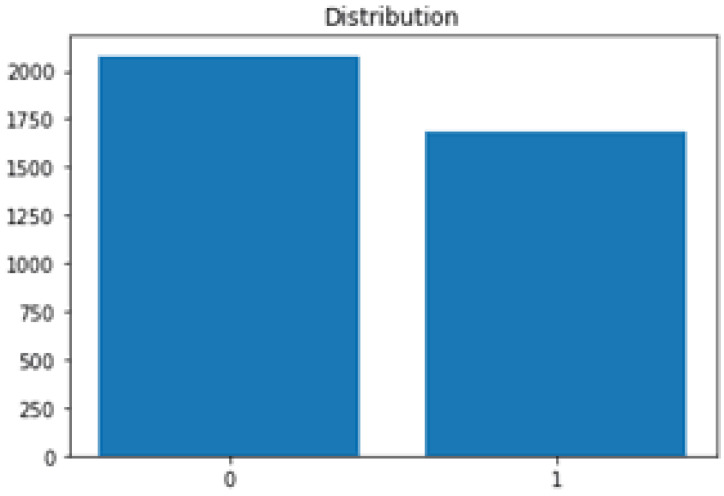
Data classification.

Figure 15 shows the pictorial view of the whole dataset, in which 2079 images had no tumor and 1683 images included tumors. These numbers represent how correctly the model predicted the tumor and normal images.

**Figure 16:**
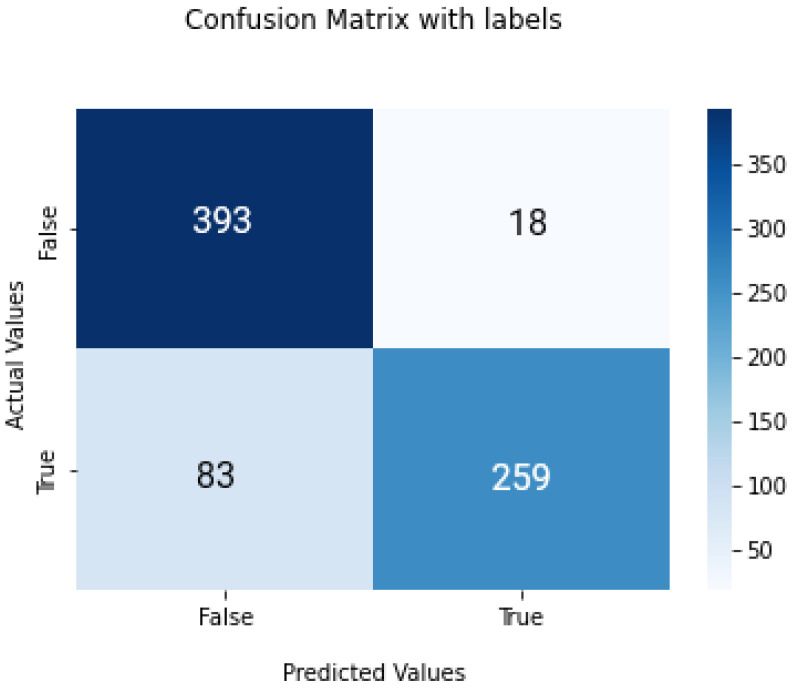
Proposed-model confusion matrix.

Figure 16 shows the confusion matrix for the result predicted by the model. A total of 111 images weer misclassified out of 753 images used for the testing phase. For the No-tumor label, the achieved F1-Score, precision and recall were 96.1%, 96.5% and 95.62%, respectively. For the tumor label, F1-Score was 96.68%, precision 96.50% and recall 95.73%.

The model was trained efficiently through the use of ResNet-50 and a 2-D convolutional layered network. The brain tumor dataset used was divided between training and testing data. Achieved accuracy for training and testing was 99.82% and 99.5%, respectively. Data preprocessing, pixel reduction, loss function, and optimizer are some of the techniques used for facilitating the experimentation phase. The performance achieved using the SGD optimizer is highly generalized and accurate. The uniqueness of this model is that it has been trained upon a large number of datasets which was previously not performed. In addition, the accuracy achieved with a combination of CNN and SGD made it a more preferable approach to adopt for the prediction of brain tumors.The limitation of using CNN with SGD is that the model is trained using a very small learning rate. To further enhance the efficiency of the model and make it more generalized, the proposed model should be trained with stochastic gradient classifiers other than SGD.

The results of the proposed method were also compared with some state-of-the-art methods in terms of accuracy and F1-Score. It is evident that the proposed method outperformed the baseline methods. The significance of the diagnosis provided by the doctor increased the accuracy of their assistance in identifying the tumour and treating the patient. The comparative results are presented in Table 5.

## 6. Conclusions

This paper focused on developing a general model that works best for the detection of tumors in brain cells. The major objective behind conducting this research was to help doctors identify one of most precarious diseases found in humans. The dataset used for this research was taken from the Kaggle repository. In total, there are 3762 images in the dataset. This study used the CNN deep-learning algorithm to classify tumors. ResNet-50 and a 2D layered network of CNN were utilized to train the model. SGD optimizer algorithm was deployed to enhance the performance of the model. The accuracy observed during the training phase came out to be 99.82% and the accuracy achieved during the model testing phase was 99.5%. Techniques of computer vision (CV) such as image processing and image segmentation were used to facilitate the research work. The performance of the model achieved using the SGD optimizer is highly generalized and precise. In the future, the accuracy and some other performance-measure values can be improved using a large number of datasets with some other deep-learning approaches utilizing an SGD optimizer or using CNN with gradient-boosting algorithms other than SGD. Besides using more datasets for achieving more improved accuracy from the system, the research can also progress by developing approaches to specifically classify tumors on the basis of their characteristics or by their disease type, i.e., malignant or benign.

## Figures and Tables

**Table 1:** Prediction algorithm as applied in EDM.

Ref.	Algorithm	Dataset	Results
[32]	3D deep neural network	BraTS 2017	Detected and segmented brain tumor into the core, whole, and magnified tumor with a coefficient of 0.69,0.81 & 0.55, respectively.
[33]	Modified genetic algorithm, support vector machine	BRATS 2018 & BRATS 2019	Classified the tumor with accuracy of 99.7% and 98.8% for BRATS 2018 and 99.8% and 99.3% for 2019.
[34]	Darknet-53 YOLOv3 algorithm (deep neural network-based)	MSCOCO (Microsoft Common Objects in Context) data set and final image data set	The position of the tumor in the head was detected effectively with amplified accuracy and diminished validation loss.
[20]	Standard balanced digital link (SBDL) and particle swarm optimization (PSO)	MRI imagres dataset	At classifying brain tumors, achieved more than 92% accuracy.
[35]	Discrete wavelet transform (DWT), support vector machine (SVM)	Brain MRI images	SVM proves to be a robust algorithm for the recognition of brain tumors, with 94.6% accuracy.
[36]	Fuzzy C-means clustering with super-resolution (FCM-SR) & CNN	Cancer Genome Atlas Glioblastoma Multiform (TCGA-GBM) database	FCM-SR recognized brain tumor with a magnificent accuracy of 98.33%
[4]	Non-dominated sorted genetic algorithm (NSGA)	BRATS 2018–2020	Brain tumors were classified and segmented accurately with final score accuracy of more than 0.90.
[24]	PSO algorithm, ANN	RIDER and BRATS 2018	99% accuracy was achieved with the proposed model for tumor identification in the brain.
[14]	Convolutional neural network (CNN)	MRI brain imageries	96.08% accuracy for three-layer CNN-trained model.
[22]	Support vector machine (SVM), Long-Short Term Memory (LSTM)	GigaDB and BCI Competition IV	OPTICAL predictor along with SVM classified motor images accurately.
[15]	Convolutional neural network (CNN)	Free Accessible MRI images	BrainMRNet model effectively identified tumor with an accuracy of 96.05 and proved better than the previously existing models.
[21]	Artificial neural network (ANN), Convolutional Neural Network (CNN)	Brain MRI Images	CLM came out with 96% accuracy while identifying the tumors.

**Table 2:** Hyperparameter configuration after performing the optimization for brain-tumor detection.

Hyperparameter	Configuration
Optimizer	SGD
Number of Epochs	50
Learning Rate	0.001
Momentum	0.9
No. of Layers	7
Filter Size	5 × 5
Batch Size	32
Activation Function	Relu

**Table 3:** Confusion matrix.

		Predicted	
		Positive	Negative
Actual	Positive	*TP*	*FN*
	Negative	*FP*	*TN*

**Table 4:** Performance measure for various techniques for brain-tumor detection.

Method	F1-Score	Precision	Recall	Accuracy
SVM	90.80%	90.12%	89.67%	91.63%
CNN	92.75%	92.90%	91.13%	93.53%
AlexNet	92.60%	93.30%	91.05%	94.50%
ResNet-18	93.33%	94.55%	90.65%	95.06%
ResNet-34	94.22%	94.64%	93.22%	96%
ResNet-50	95.05%	94.65%	91.25%	96.75%
VGG-16	95.35%	95.10%	92.95%	97.80%
Proposed Method	96.10%	96.50%	95.62%	99%

**Table 5:** Comparative result analysis.

Method	Dataset (No. of Images)	Accuracy	F1-Score
VGG-19 with ADAS Optimizer	Brain MRI Dataset (1307 Images )	94.56%	94.90%
Discrete Wavelet Transform (DWT) and support vector machine (SVM)	DICOM dataset (750 samples)	94.6%	93.56%
AlexNet with Shallow CNN and ADAM optimizer	Brain MRI Images (253 Images)	96.05%	94.12%
Fuzzy C-means with super-resolution and CNN with ADAM	The Cancer Imaging Archive (500 samples)	98.33%	-
Modified genetic algorithm and support vector machine	BRATS-2018	98.67%	93.67%
**Proposed Method**	Kaggle Br35H Dataset (804 Images)	99%	96.1%

## Data Availability

Not applicable.
